# Children with life-limiting conditions in paediatric intensive care units: a national cohort, data linkage study

**DOI:** 10.1136/archdischild-2017-312638

**Published:** 2017-07-13

**Authors:** Lorna K Fraser, Roger Parslow

**Affiliations:** 1 Department of Health Sciences, University of York, York, UK; 2 Division of Epidemiology and Biostatistics, Leeds Institute of Cardiovascular and Metabolic Medicine, University of Leeds, Leeds, UK

**Keywords:** PICU, Life-Limiting Conditions, Palliative Care, Survival, Child

## Abstract

**Objective:**

To determine how many children are admitted to paediatric intensive care unit (PICU) with life-limiting conditions (LLCs) and their outcomes.

**Design:**

National cohort, data-linkage study.

**Setting:**

PICUs in England.

**Patients:**

Children admitted to a UK PICU (1 January 2004 and 31 March 2015) were identified in the Paediatric Intensive Care Audit Network dataset. Linkage to hospital episodes statistics enabled identification of children with a LLC using an International Classification of Diseases (ICD10) code list.

**Main outcome measures:**

Random-effects logistic regression was undertaken to assess risk of death in PICU. Flexible parametric survival modelling was used to assess survival in the year after discharge.

**Results:**

Overall, 57.6% (n=89 127) of PICU admissions and 72.90% (n=4821) of deaths in PICU were for an individual with a LLC.

The crude mortality rate in PICU was 5.4% for those with a LLC and 2.7% of those without a LLC. In the fully adjusted model, children with a LLC were 75% more likely than those without a LLC to die in PICU (OR 1.75 (95% CI 1.64 to 1.87)).

Although overall survival to 1 year postdischarge was 96%, children with a LLC were 2.5 times more likely to die in that year than children without a LLC (OR 2.59 (95% CI 2.47 to 2.71)).

**Conclusions:**

Children with a LLC accounted for a large proportion of the PICU population. There is an opportunity to integrate specialist paediatric palliative care services with paediatric critical care to enable choice around place of care for these children and families.

**What is already known on this topic?**

- The prevalence of children and young people with life-limiting conditions (LLCs) or life-threatening conditions is rising.
- Overall mortality in paediatric intensive care unit (PICU) is decreasing.

**What this study adds?**

- Children with a LLC accounted for the majority of admissions, bed-days and deaths in PICU.
- Children with a LLC were75% more likely to die in PICU than those without a LLC.
- There was 93% survival at 1 year for children with a LLC.

## Introduction

Life-limiting conditions (LLCs) are those for which there is no reasonable hope of cure and from which children will ultimately die, for example, Duchenne muscular dystrophy or neurodegenerative disease. Life-threatening conditions (LTCs) are those for which curative treatment may be feasible but can fail, for example, cancer. LLC will be used to include life-limiting conditions and LTCs.

The prevalence of children and young people with a LLC is increasing^1^ partly due to more aggressive treatment of complications and the use of medical technologies, including paediatric intensive care unit (PICU). These children often have repeated hospital admissions^2^ and use increasing amounts of hospital resources.^3–5^ Many of these children also die on PICU^6^ when treatment fails or is withdrawn. This study aims to ascertain what proportion of admissions to PICUs are for children with a LLC and their outcomes in PICU and up to 1 year postdischarge.

## Methods

### Datasets

The Paediatric Intensive Care Audit Network (PICANet) collects data on all children admitted to PICUs in the UK and Ireland. All admissions to a PICU in the UK between 1 January 2004 and 31 March 2015 were identified in the PICANet dataset.^7^ Only children resident in England were included as only their inpatient hospital data (Hospital Episodes Statistics (HES)) were available for linkage.^8^ Hospital data for the other nations of the UK were not available.

The Office for National Statistics (ONS) death record data in England were available with a censor date of 1 November 2015.^9^

Linkage of the PICANet dataset to the HES and ONS data was undertaken by the NHS Digital.^10^ The standard deterministic linkage algorithm using National Health Service (NHS) number, date of birth, sex and postcode was used.

### Clinical variables

#### Inpatient HES data

The PICANet data are of high quality and validated, but some of the non-mandatory fields, including comorbidities, are incomplete. Therefore, it is not possible to identify children with a LLC using the PICANet dataset alone. Linkage to the inpatient HES data (1 April 1997 to 31 March 2015) enabled the use of a previously developed International Classification of Diseases (ICD10) coding framework^1^ to identify individuals with a LLC (see online supplementary table 1). A PICU admission was categorised with a LLC if one of the LLC codes were recorded within the HES data for that individual before the date of PICU discharge. For the analyses for survival in the year after PICU discharge, LLC codes up to the censor date were included.

10.1136/archdischild-2017-312638.supp1 Supplementary Tables

#### PICANet data

Clinical diagnoses were coded using clinical terms 3 and aggregated into 12 primary diagnostic groups.^11^

Risk adjustment for mortality used the log odds of mortality based on the Paediatric Index of Mortality 2 (PIM2) with recalibrated coefficients calculated using data from 2011 to 2013.^12^ PIM2 was categorised into five categories of risk: <1%, 1 to <5%, 5% to <15%, 15% to <30% and 30%+.

Length of stay was categorised into <1, 1 to <3, 3 to <7, 7 to <14, 14 to <28 and ≥28 days. The total number of bed-days for each individual was calculated for all their PICU admissions. The number of PICU admissions were categorised as single admission, two admissions, three admissions and four or more admissions.

The type of admission was defined as planned after surgery, unplanned after surgery, planned other and unplanned.

#### ONS death data

Date of death was obtained from the ONS data.^9^

### Sociodemographic variables

Age at admission to PICU was categorised as <1, 1–4, 5–10, 11–15 and ≥16 years. Sex was included in the analysis only where it was non-ambiguous.

An Index of Multiple Deprivation^13^ category was assigned to each individual based on their lower super output area (LSOA) of residence. An LSOA is a census geographical area built up of output areas with population of 1000–3000 per LSOA.^14^

Ethnicity is poorly recorded in all the datasets; therefore, ethnicity was determined using two name analysis programmes which classified children as South Asian (Pakistani, Indian, Bangladeshi): Nam Pehchan^15 16^ and the South Asian Names and Group Recognition Algorithm.^17^ The results were corrected manually for known misclassification errors.^18^ Ethnicity was assessed as South Asian or not, as the South Asian population are the largest minority ethnic group in the UK.^19^

### Statistical analyses

Descriptive statistics were undertaken, and differences between groups were assessed by χ^2^ or t-test.

Random-effects logistic regression was undertaken to account for inter-PICU variation in the outcome, death in PICU. Variables were included via a forced entry method and retained if p<0.05 or if they improved the model fit assessed using the Bayesian information criterion (BIC).

Flexible parametric survival modelling was undertaken to assess survival in the year after discharge from PICU rather than traditional Cox regression as the proportional hazards assumption was violated.^20^ Data from the last PICU admission for each individual discharged alive from PICU were included.

Analyses were carried out using STATA V.13, and tests of statistical significance were at p≤0.05.

### Ethics approval

Collection of personally identifiable data has been approved by the Patient Information Advisory Group (now the Health Research Authority Confidentiality Advisory Group), and ethics approval was granted by the Trent Medical Research Ethics Committee (ref. 05/MRE04/17 +5).

## Results

### Cohort and linkage

**Figure 1:**
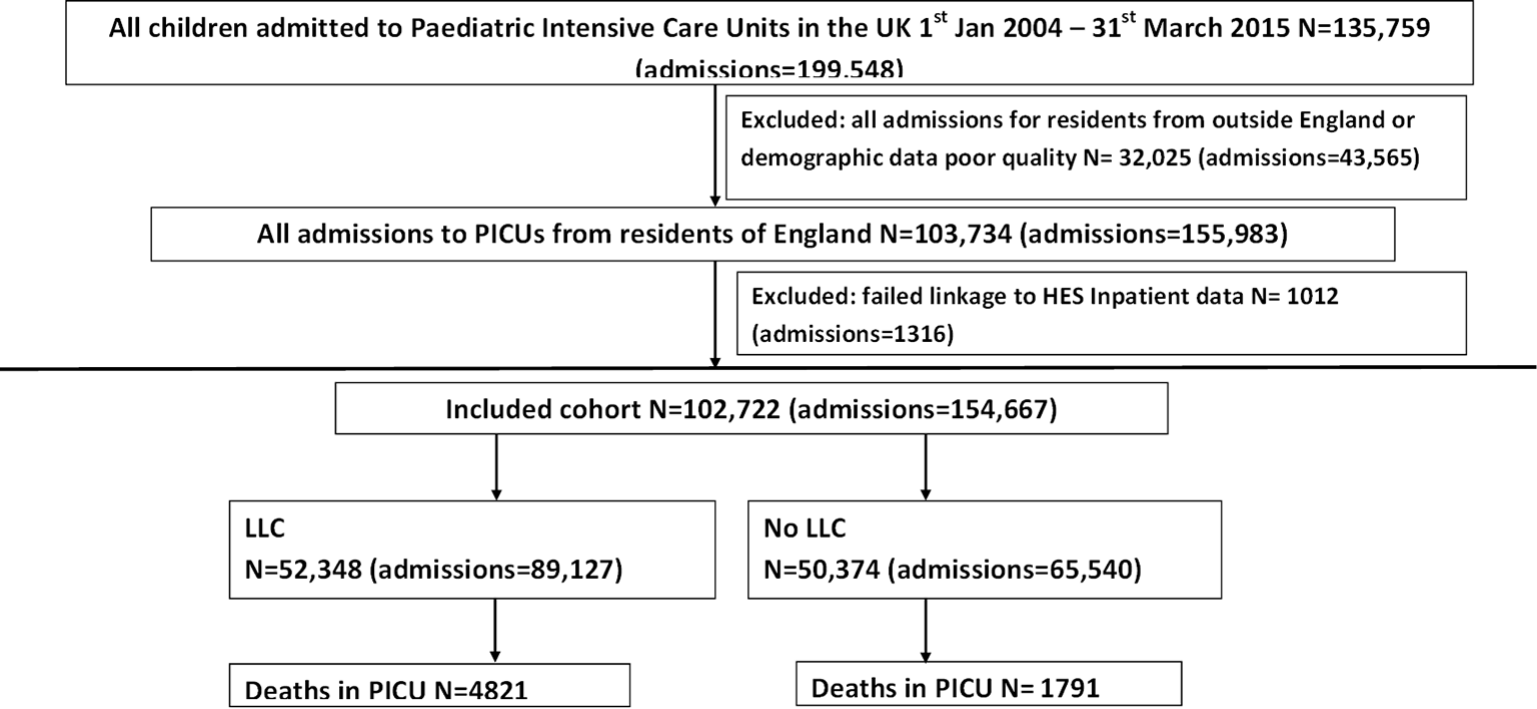
Study flowchart. HES, Hospital Episodes Statistics; LLC, life-limiting condition; PICU, paediatric intensive care unit.

Nearly 200 000 PICU admissions occurred in the UK in the study period. After excluding non-English residents and those with poor-quality demographic data (denoting missing NHS number and date of birth which are required for linkage), data for 103 374 individuals were sent for linkage. Linkage was successful for 102 722 individuals (99%) who had 154 667 PICU admissions (figure 1).

There were no significant differences between those who linked and those in whom linkage was not successful by sex, ethnicity, PIM2 score or length of PICU stay (see online supplementary table 2). Some significant differences were found; linkage improved from 98.0% in 2004 to 99.4% in 2015 (χ^2^=365, p<0.001), fewer >16-year-olds linked compared with the <1-year-olds (98.9% vs 99.3%) and children with more PICU admissions were more likely to be linked than those with a single admission (99.5% vs 98.9%, χ^2^=120, p<0.001).

### Descriptive statistics

Overall, 57.6% (n=89 127) of PICU admissions were for an individual with a LLC (table 1). Excluding 2015 data which in only part year, the percentage of admissions to PICU for those with a LLC has increased from 51.8% to 61.0%. There was a U-shaped association with age with 58.5% of the <1-year-olds admitted to PICU having a LLC, 50.2% of the children aged 11- to 15 years and 65.4% of the >16-year-olds. More of the admissions from children with a South Asian background had a LLC compared with non-South Asians (62.9% vs 56.9% χ^2^=233, p<0.001).

**Table 1:** Descriptive statistics of PICU admissions by LLC status (with row %)

	Total	LLC	%	No LLC	%	Χ^2^	p Value
Number	154 667	89 127	57.6	65 540	42.4		
Age category						556	<0.001
<1 year	72 170	42 232	58.5	29 938	41.5		
1–4 years	39 571	23 097	58.4	16 474	41.6		
5–10 years	20 448	11 982	58.6	8466	41.4		
11–15 years	19 003	9542	50.2	9461	49.8		
16+	3467	2267	65.4	1200	34.6		
Missing	8	7		1			
Sex						3.1	0.21
Male	87 686	50 422	57.5	37 264	42.5		
Female	66 933	38 682	57.8	28 251	42.2		
Missing	48	23		25			
Ethnicity						233	<0.001
Non-South Asian	1 36 670	77 804	56.9	58 866	43.1		
South Asian	17 997	11 323	62.9	6674	37.1		
Deprivation category						74.7	<0.001
Category 1 (least deprived)	21 421	12 101	56.5	9320	43.5		
Category 2	21 816	12 573	57.6	9243	42.4		
Category 3	26 341	15 437	58.6	10 904	41.4		
Category 4	34 498	19 935	57.8	14 563	42.2		
Category 5 (most deprived)	49 538	28 361	57.3	21 177	42.7		
Missing	1053	720		333			
Diagnostic group (reason for PICU admission)						1300	<0.001
Neurological	17 270	8154	47.2	9116	52.8		
Cardiac	44 767	32 465	72.5	12 302	27.5		
Respiratory	42 230	21 687	51.4	20 543	48.6		
Oncology	5190	4663	89.8	527	10.2		
Infection	8014	3468	43.3	4546	56.7		
Musculoskeletal	5736	3192	55.6	2544	44.4		
Gastrointestinal	10 019	5245	52.4	4774	47.6		
Other	8140	4554	55.9	3586	44.1		
Blood and lymph	1456	757	52.0	699	48.0		
Trauma	4581	405	8.8	4176	91.2		
Endocrine/metabolic	3878	2131	55.0	1747	45.0		
Multisystem	427	402	94.1	25	5.9		
Body wall and cavities	2959	2004	67.7	808	32.3		
Risk of mortality (PIM2)						2001	<0.001
<1%	48 957	25 583	52.3	23 374	47.7		
1% to <5%	74 212	42 403	57.1	31 809	42.9		
5% to <15%	24 727	16 261	65.8	8466	34.2		
15% to <30%	4270	3321	77.8	949	22.2		
>30%	2501	1559	62.3	942	37.7		
LOS PICU (days)						5600	<0.001
<1	45 246	22 420	49.6	22 826	50.4		
1 to <3	49 285	26 579	53.9	22 706	46.1		
3 to <7	34 122	20 381	59.7	13 741	40.3		
7 to <14	15 957	11 342	71.1	4615	28.9		
14 to <28	6603	5401	81.8	1202	18.2		
28+	3412	2986	87.5	426	12.5		
Missing	42	18	42.9	24	57.1		
Type of PICU admission						3600	<0.001
Planned, after surgery	49 749	33 034	66.4	16 715	33.6		
Unplanned, after surgery	7688	3985	51.8	3703	48.2		
Planned other	10 900	7551	69.3	3349	30.7		
Unplanned	86 050	44 412	51.6	41 638	48.4		
Not known	280	145		135			
Year of PICU admission						574	<0.001
2004	12 293	6366	51.8	5927	48.2		
2005	12 326	6531	53.0	5795	47.0		
2006	12 634	7116	56.3	5518	43.7		
2007	13 275	7492	56.4	5783	43.6		
2008	13 462	7463	55.4	5999	44.6		
2009	14 023	7994	57.0	6029	43.0		
2010	14 185	8341	58.8	5844	41.2		
2011	14 006	8282	59.1	5724	40.9		
2012	14 597	8904	61.0	5693	39.0		
2013	14 865	9126	61.4	5739	38.6		
2014	14 973	9137	61.0	5836	39.0		
2015	4028	2375	59.0	1653	41.0		

LLC, life-limiting condition; LOS, length of stay; PICU, paediatric intensive care unit; PIM2, Paediatric Index of Mortality 2.

Differences between the two groups existed for the clinical variables with 94.1% of those children whose reason for PICU admission was multisystem having a LLC compared with only 8.8% of trauma cases and 43.3% of infective cases (χ^2^=1300, p<0.001).

The risk of mortality scores varied by LLC status with 52.3% of those with a PIM2 score <1% having a LLC, 77.8% of those with a PIM2 score of 15% to <30% and 62.3% of those with a PIM2 score of >30% (χ^2^=2300, p<0.001).

A linear association with length of PICU stay was shown with 49.6% of those with a PICU stay of <1 day and 87.5% of those staying in PICU >28 days having a LLC (χ^2^=6000, p<0.001). The median length of stay was 2.6 days (IQR 1.0–6.1) for those with a LLC compared with 1.6 days (IQR 0.8–3.5) for those without a LLC.

The total number of PICU bed days for this cohort was 763 664; children with a LLC accounted for 72.6% (554 404).

More than 66% of the planned PICU admissions after surgery were for children with a LLC compared with 51.6% of unplanned PICU admissions (χ^2^=3600, p<0.001).

### Deaths

A total of 11 588 children had died at the censor date, with 6612 deaths occurring in PICU. Children with a LLC accounted for 72.9% (n=4821) of PICU deaths and 87.4% (n=4397) of deaths after discharge. The crude PICU mortality rate was 5.4% for those with a LLC and 2.7% for those without a LLC.

### Death in PICU

The unadjusted risk of death in PICU for children with a LLC was nearly twice that of those without a LLC (OR 1.94 (95% CI 1.84 to 2.06)). After adjusting for expected risk of mortality and other clinical and demographic variables, children with a LLC were 75% more likely than those without a LLC to die in PICU (OR 1.75 (95% CI 1.64 to 1.87)) (table 2).

**Table 2:** Random-effects logistic regression model for death in PICU

	OR	95% CIs		p Value
LLC				
No	Ref			
Yes	1.75	1.64	1.87	<0.001
Age category				
<1 year	Ref			
1–4 years	0.81	0.75	0.87	<0.001
5–10 years	0.94	0.86	1.03	0.20
11–15 years	1.06	0.96	1.16	0.26
16+	1.37	1.13	1.66	<0.001
Sex				
Male	Ref			
Female	1.09	1.03	1.15	0.002
Ethnicity				
Non-South Asian	Ref			
South Asian	1.30	1.20	1.41	<0.001
Deprivation category				
Category 1 (least deprived)	Ref			
Category 2	1.02	0.91	1.13	0.77
Category 3	1.03	0.92	1.14	0.64
Category 4	1.07	0.97	1.18	0.18
Category 5 (most deprived)	1.07	0.97	1.17	0.17
Diagnostic group (reason for PICU admission)				
Neurological	1.39	1.26	1.54	<0.001
Cardiac	1.23	1.13	1.35	0.001
Respiratory	Ref			
Oncology	2.06	1.75	2.42	<0.001
Infection	1.94	1.74	2.17	<0.001
Musculoskeletal	0.74	0.55	0.99	0.04
Gastrointestinal	1.39	1.22	1.58	<0.001
Other	1.26	1.10	1.45	<0.001
Blood and lymph	2.32	1.86	2.91	<0.001
Trauma	1.69	1.43	2.01	<0.001
Endocrine/metabolic	2.18	1.90	2.50	<0.001
Multisystem	0.67	0.33	1.38	0.28
Body wall and cavities	0.97	0.76	1.22	0.78
Risk of mortality (PIM2)				
<1%	Ref			
1% to <5%	4.54	3.91	5.28	<0.001
5% to <15%	12.46	10.65	14.57	<0.001
15% to <30%	32.56	27.44	38.64	<0.001
>30%	201.63	169.60	239.70	<0.001
LOS PICU (days)				
<1	1.51	1.39	1.63	<0.001
1 to <3	Ref			
3 to <7	0.86	0.79	0.94	0.001
7 to <14	1.09	0.99	1.20	0.07
14 to <28	2.02	1.81	2.24	<0.001
>28	3.98	3.53	4.47	<0.001
Type of PICU admission				
Planned, after surgery	Ref			
Unplanned, after surgery	1.20	1.01	1.42	0.04
Planned other	1.32	1.14	1.52	<0.001
Unplanned	1.53	1.38	1.70	<0.001
Not known	1.35	0.63	2.88	0.44
Year of admission	0.97	0.96	0.98	<0.001

n=153 513, group=35, Wald χ^2^=10 213, BIC=40 229, sigma_u=0.30, rho=0.03.

BIC, Bayesian information criterion; LLC, life-limiting condition; LOS, length of stay; PICU, paediatric intensive care unit; PIM2, Paediatric Index of Mortality 2.

Stratified analyses by LLC status highlighted some differences between the main variables associated with a higher risk of death in PICU (see online supplementary table 3a and b). For those with a LLC, being older than age 16 years (OR 1.37 (95% CI 1.12 to 1.67)) and of South Asian origin (OR 1.30 (95% CI 1.20 to 1.41)) had a higher risk of death. This was not seen for those without a LLC. The diagnoses with highest risk of death in PICU were blood and lymph (OR 2.54 (95% CI 1.98 to 3.25)) or endocrine/metabolic (OR 2.38 (95% CI 2.05 to 2.76)) for those with a LLC compared with trauma (OR 2.37 (95% CI 1.84 to 3.00)) or neurological conditions (OR 2.19 (95% CI 1.79 to 2.69)) for those without a LLC. The risk of death was highest for stays longer than 7 days in those with a LLC but not until 14 days for those without a LLC.

The odds of dying in PICU decreased by 3% each year (OR 0.98 (95% CI 0.97 to 0.99)).

### Survival after discharge from PICU

**Figure 2:**
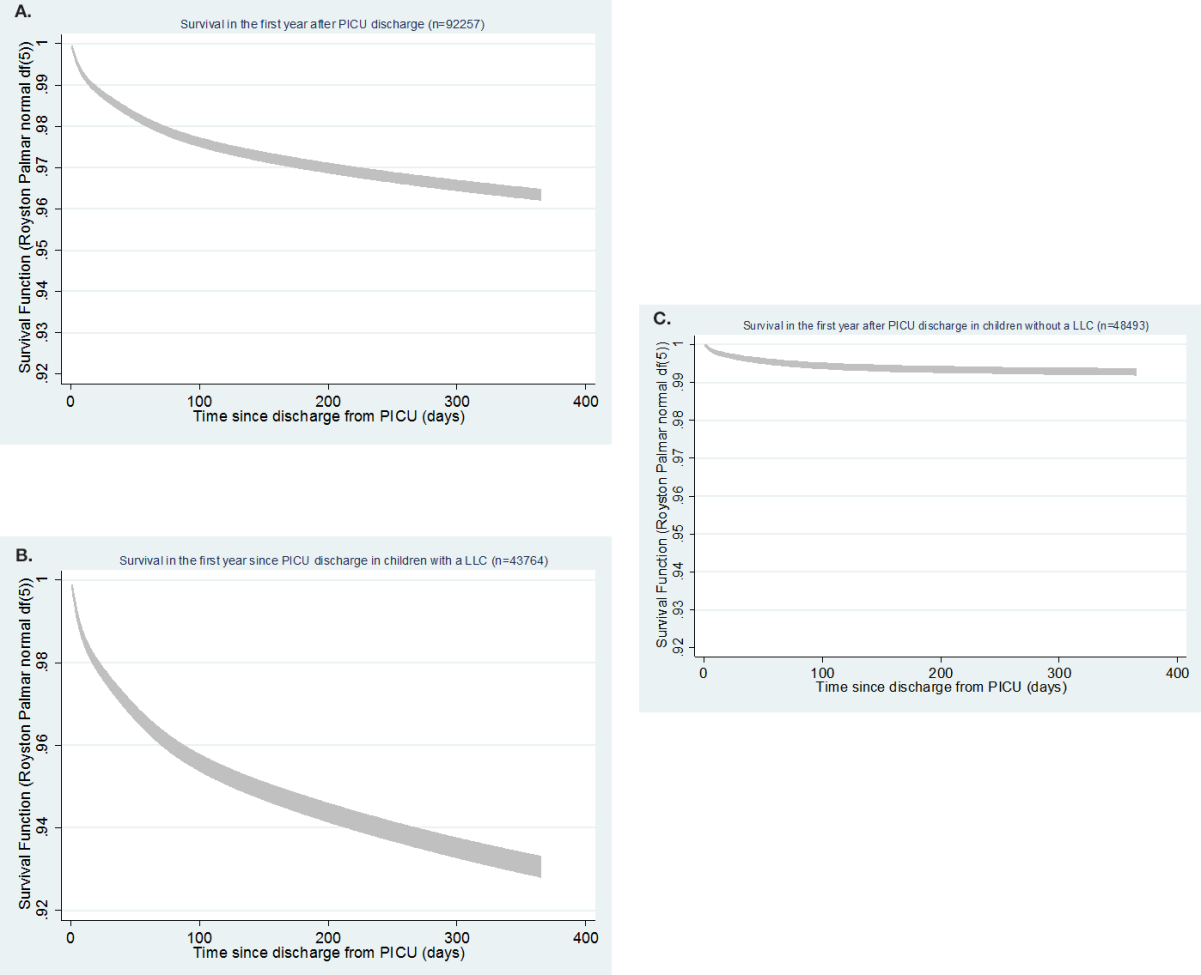
Survival curves with 95% CIs. LLC, life-limiting condition; PICU, paediatric intensive care unit.

Overall survival rate is >96% at 1 year after PICU (figure 2A). There are differences between these survival functions for children with (figure 2B) and without a LLC (figure 2C). There is a steeper curve in the first 3 months after discharge from PICU for those with a LLC with approximately 93% still alive at 1 year postdischarge. For those without a LLC, the survival curve is much flatter, and >99% are alive at 1 year post-PICU discharge.

A log normal distribution model with 5 df provided the best fit assessed using BIC (table 3). There are some similarities to the death in PICU model: children with a LLC (OR 2.59 (95% CI 2.47 to 2.71)), those from a South Asian background (OR 1.19 (95% CI 1.13 to 1.25)) and those from the most deprived category (OR 1.08 (95% CI 1.02 to 1.14) were more likely to die in the year after discharge from PICU than children without a LLC, non-South Asian and those in the least deprived areas, respectively. All other types of PICU admission had significantly higher odds of death compared with the planned after surgery group and the odds of dying after discharge decreased by 3% with each increasing year of admission (OR 0.97 (95% CI 0.96 to 0.98)). Compared with the reference group of respiratory reasons for PICU admission, those with an oncology (OR 1.83 (95% CI 1.70 to 1.97)) or neurology diagnoses (OR 1.17 (95% CI 1.11 to 1.24)) were more likely to die in the year after discharge from PICU. Those with trauma (OR 0.63 (95% CI 0.53 to 0.77)) or body wall and cavities (OR 0.63 (95% CI 0.54 to 0.72)) diagnoses were significantly less likely to die in the year after discharge from PICU.

**Table 3:** Results of flexible parametric survival modelling for survival to 365 days after discharge from PICU

	HR	95% CIs		p Value
Age category				
<1 year	Ref			
1–4 years	0.83	0.80	0.87	<0.001
5–10 years	0.77	0.73	0.82	<0.001
11–15 years	0.85	0.80	0.90	<0.001
16+	0.98	0.89	1.09	0.72
Sex				
Male	Ref			
Female	1.02	0.99	1.06	0.23
Ethnicity				
Non-South Asian	Ref			
South Asian	1.19	1.13	1.25	<0.001
Deprivation category				
Category 1 (least deprived)	Ref			
Category 2	0.99	0.92	1.05	0.66
Category 3	1.03	0.96	1.09	0.41
Category 4	1.06	1.00	1.13	0.04
Category 5 (most deprived)	1.08	1.02	1.14	0.01
LLC				
No	Ref			
Yes	2.59	2.47	2.71	<0.001
Diagnostic group (reason for PICU admission)				
Neurological	1.17	1.11	1.24	<0.001
Cardiac	0.86	0.81	0.90	<0.001
Respiratory	Ref			
Oncology	1.83	1.70	1.97	<0.001
Infection	0.87	0.80	0.94	0.001
Musculoskeletal	0.91	0.81	1.03	0.152
Gastrointestinal	1.04	0.97	1.12	0.276
Other	1.04	0.96	1.13	0.339
Blood and lymph	0.98	0.82	1.17	0.79
Trauma	0.63	0.53	0.77	<0.001
Endocrine/metabolic	1.08	0.98	1.20	0.117
Multisystem	0.97	0.70	1.33	0.831
Body wall and cavities	0.63	0.54	0.72	<0.001
Risk of mortality (PIM2)				
<1%	Ref			
1% to <5%	1.28	1.22	1.35	<0.001
5% to <15%	1.55	1.45	1.64	<0.001
15% to <30%	2.07	1.88	2.28	<0.001
>30%	2.46	2.12	2.85	<0.001
LOS PICU (days)				
<1	1.14	1.08	1.19	<0.001
1 to <3	Ref			
3 to <7	1.06	1.01	1.12	0.01
7 to <14	1.29	1.22	1.37	<0.001
14 to <28	1.58	1.47	1.71	<0.001
>28	1.75	1.57	1.95	<0.001
Type of PICU admission				
Planned, after surgery	Ref			
Unplanned, after surgery	1.22	1.12	1.33	<0.001
Planned other	1.65	1.54	1.78	<0.001
Unplanned	1.37	1.29	1.44	<0.001
Not known	1.17	0.76	1.78	0.48
Year of admission	0.97	0.96	0.98	<0.001

n=91 614.

HR, hazard ratio; LLC, life-limiting condition; LOS, length of stay; PICU, paediatric intensive care unit; PIM2, Paediatric Index of Mortality 2.

In contrast to the in-PICU death models, all those aged 1–15 years were significantly less likely to die than the <1 age group.

## Discussion

Children with a LLC accounted for nearly 58% of all admissions to PICU, 72% of PICU bed-days and 87.5% of all PICU admissions that lasted >28 days. Although the mortality rate continues to decrease in PICU, 73% of all deaths in PICU during this study were in children with a LLC. The survival in the year after PICU discharge was also significantly lower in children with a LLC compared with those without a LLC.

The high number and percentage of PICU admissions for children with a LLC is similar to results from a US study in which children with complex chronic conditions (CCCs) accounted for 53% (range 22.4%–70.6%) of PICU admissions.^21^ The definitions used to identify the populations with CCCs were different to the LLC definition used in the current study. A multicountry prevalence study found that 67% of children had a CCC or disability within PICU or neonatal intensive care unit.^22^

Previous work has found that children with a CCC had an increased risk of prolonged length of PICU stay (>15 days)^21^ and children who died in PICU have longer lengths of stay before death.^23^ This study has shown that the risk of death in PICU is significantly higher for those with a LLC who have been in PICU for longer than 7 days.

The higher PICU crude death rate for children with a LLC is not unexpected and confirms the patterns seen in the US study where they found in-PICU mortality of 3.9% for those with a CCC compared with 2.2% for children with no chronic condition and 0.3% for those with non-CCCs.^21^ However, death in a child with a LLC may be expected, and admissions to PICU are known to be stressful^24–27^ and parents and siblings of children who died in hospital show more psychological symptoms^28^ and poorer adjustment^29^ than if their child had died at home. If the child is likely to die despite PICU admission, then an alternative place of care such as being cared for at home or in a hospice by specialist paediatric palliative care may be more appropriate. Guidance from The European Association of Palliative Care^30^ and the International Children’s Palliative Care Network^31^ both state that the family home should, where possible, be the main place of care and that these families should have access to paediatric palliative care services.

With in-PICU mortality falling to low levels, it is important that other in-/post-PICU outcomes such as quality of life or functional status are assessed, especially for this group of children with high-care needs.

Although the vast majority of children survived their PICU admission, nearly 7% of those with a LLC will die in the year after discharge from PICU with many of these deaths occurring in the first 3 months. PICU staff are highly experienced at caring for a dying child and their family, but given the expansion of specialist paediatric palliative care services and the children’s hospice sector over the last decade, further integration of these services may offer the family more choice over place of care or death for their child and can often offer longer term input, both when the child has died and in the bereavement period than is possible from a PICU.

### Strengths/limitations

This is the first national study providing data on survival following PICU admission in this population of children, and it used linked audit, administrative and hospital data. Identification of children with a LLC in this dataset was via the HES data. This is an administrative dataset in which the coding has improved over time, but its primary aim is not as a research dataset. Lack of agreement on definitions of some complex conditions has been shown previously.^32^ Having complete data for comorbidities in the PICANet dataset, which is audited for quality, would be preferable.

### Conclusions

Children with a LLC accounted for nearly 58% of admissions to PICU, 72% of bed-days, 87.5% of stays greater than 28 days and 73% of deaths in PICU. There is an opportunity, given the recent growth in specialist paediatric palliative care services, to have integration of these services to enable choice around place of care and place of death for these children and families.

Future studies collecting high-quality information on changes in functional status and quality of life are vital to further gauge the clinical value of these PICU admissions.
