# Supplementary material for: Children with life-limiting conditions in paediatric intensive care units: a national cohort, data linkage study
Source: Arch Dis Child. 2017 Jul 13;103(6):540–7. doi: 10.1136/archdischild-2017-312638 (PMC5965357; doi:10.1136/archdischild-2017-312638)
Supplement: Supplementary Tables [file archdischild-2017-312638supp001.pdf]

Supplemental Table 1 ICD10 FILTER CODES to IDENTIFY LIFELIMTING DIAGNOSES IN CHILDREN & YOUNG PEOPLE

**A**	**B**	**C**	**D**	**E**	**F**	**G**	**G**	**H**	**I**	**J**
A17<br>A81.0<br>A81.1	B20-B24	C00-C97	D33<br>D43<br>D44.4<br>D48<br>D56.1<br>D61.0<br>D61.9<br>D70<br>D76.1<br>D81<br>D82.1<br>D83<br>D89.1	E31.0<br>E34.8<br>E70.2<br>E71<br>E72<br>E74<br>E75<br>E76<br>E77<br>E79.1<br>E83.0<br>E84<br>E88.0<br>E88.1	F80.3<br>F84.2	G10<br>G11.1<br>G11.3<br>G12<br>G20<br>G23.0<br>G23.8<br>G31.8<br>G31.9<br>G35<br>G40.4<br>G40.5<br>G60.0<br>G60.1<br>G70.2<br>G70.9<br>G71.0<br>G71.1<br>G71.2<br>G71.3<br>G80.0<br>G80.8	G82.3<br>G82.4<br>G82.5<br>G93.4<br>G93.6<br>G93.7	H11.1<br>H49.8<br>H35.5	I21<br>I27.0<br>I42<br>I61.3<br>I81	J84.1<br>J96<br>J98.4
**K**	**L**	**N**	**P**	**Q**	**Q**	**Q**	**Q**	**Q**	**T**	**Z**
K55.0<br>K55.9<br>K72<br>K74<br>K76.5<br>K86.8	M31.3<br>M32.1<br>M89.5	N17<br>N18<br>N19<br>N25.8	P10.1<br>P11.2<br>P21.0<br>P28.5<br>P29.0<br>P29.3<br>P35.0<br>P35.1<br>P35.8<br>P37.1	Q00.0<br>Q01<br>Q03.1<br>Q03.9<br>Q04.0<br>Q04.2<br>Q04.3<br>Q04.4<br>Q04.6<br>Q04.9	Q21.8<br>Q22.0<br>Q22.1<br>Q22.4<br>Q22.5<br>Q22.6<br>Q23.0<br>Q23.4<br>Q23.9<br>Q25.4	Q39.6<br>Q41.0<br>Q41.9<br>Q43.7<br>Q44.2<br>Q74.8<br>Q44.5<br>Q44.7<br>Q60.1<br>Q60.6	Q78.0<br>Q78.5<br>Q79.2<br>Q79.3<br>Q80.4<br>Q81<br>Q82.1<br>Q82.4<br>Q85.8<br>Q86.0	Q93.2<br>Q93.3<br>Q93.4<br>Q93.5<br>Q93.8<br>Q95.2	T86.0<br>T86.2	Z51.5

			P52.4<br>P52.5<br>P52.9<br>P83.2<br>P91.2<br>P91.6<br>P96.0	Q07.0<br>Q20.0<br>Q20.3<br>Q20.4<br>Q20.6<br>Q20.8<br>Q21.3<br>Q23.2	Q25.6<br>Q26.2<br>Q26.4<br>Q26.8<br>Q28.2<br>Q32.1<br>Q33.6	Q61.4<br>Q61.9<br>Q64.2<br>Q74.3<br>Q75.0<br>Q77.2<br>Q77.3<br>Q77.4	Q87.0<br>Q87.1<br>Q87.2<br>Q87.8<br>Q91<br>Q92.0<br>Q92.1<br>Q92.4<br>Q92.7			

Supplemental Table 2 Comparison of descriptive statistics between those PICU admissions which did and did not Link to the HES

	Linked		Linkage unsuccessful		x^2	P value
**Number**	154,667	*99.2*	1316	*0.8*		
**Age Category**					25.5	<0.001
<1 year	72,170	*99.3*	533	*0.7*		
1-4 years	39,571	*99.1*	347	*0.9*		
5-10 years	20,448	*99.0*	214	*1.0*		
11-15 years	19,003	*99.0*	184	*1.0*		
16+	3,467	*98.9*	38	*1.1*		
missing	8	*100.0*	0	*0.0*		
**Sex**					0.43	0.51
Male	87,686	*99.2*	726	*0.8*		
Female	66,933	*99.1*	575	*0.9*		
missing	48		15			
**Ethnicity**					2.6	0.10
Non South Asian	136,670	*99.2*	1,144	*0.8*		
South Asian	17,997	*99.1*	172	*0.9*		
**Deprivation Category**					148	<0.001
Category 1 (least deprived)	21,421	*99.3*	152	*0.7*		
Category 2	21,816	*98.8*	268	*1.2*		
Category 3	26,341	*98.9*	284	*1.1*		
Category 4	34,498	*99.2*	272	*0.8*		
Category 5 (most deprived)	49,538	*99.5*	230	*0.5*		
missing	1,053	*90.5*	110	*9.5*		
**Diagnostic Group (reason for PICU admission)**					184	<0.001
Neurological	17,270	*99.0*	181	*1.0*		
Cardiac	44,767	*99.4*	268	*0.6*		
Respiratory	42,230	*99.3*	279	*0.7*		
Oncology	5,190	*98.4*	84	*1.6*		
Infection	8,014	*99.1*	73	*0.9*		
Musculoskeletal	5,736	*98.9*	61	*1.1*		
Gastrointestinal	10,019	*98.9*	112	*1.1*		
Other	8,140	*98.7*	109	*1.3*		
Blood and lymph	1,456	*98.3*	25	*1.7*		
Trauma	4,581	*98.3*	77	*1.7*		
Endocrine/metabolic	3,878	*99.2*	32	*0.8*		
Multisystem	427	*99.5*	2	*0.5*		
Body wall and cavities	2,959	*99.6*	13	*0.4*		
**Risk of Mortality (PIM 2)**					8.8	0.07
<1%	48,957	*99.2*	415	*0.8*		
1-<5%	74,212	*99.1*	668	*0.9*		
5-<15%	24,727	*99.3*	185	*0.7*		
15-<30%	4,270	*99.4*	25	*0.6*		
>30%	2,501	*99.1*	23	*0.9*		
**LOS PICU (days)**					10.5	0.11
<1	45,246	*99.1*	426	*0.9*		
1 to <3	49,285	*99.2*	413	*0.8*		

3 to <7	34,122	*99.2*	273	*0.8*		
7 to <14	15,957	*99.3*	120	*0.7*		
14 to <28	6,603	*99.3*	48	*0.7*		
28+	3,412	*99.0*	36	*1.0*		
missing	42		0			
**Type of PICU admission**					45.5	<0.001
Planned - after surgery	49749	*99.2*	398	*0.79*		
Unplanned after surgery	7688	*99.2*	64	*0.83*		
Planned other	10900	*98.6*	155	*1.40*		
Unplanned	86050	*99.2*	696	*0.80*		
Not known	280		3			
**No. PICU admissions**					120	<0.001
one admission	77,426	*98.9*	837	*1.1*		
2 admissions	28,850	*99.2*	238	*0.8*		
3 admissions	15,726	*99.4*	90	*0.6*		
4+ admissions	32,665	*99.5*	151	*0.5*		
**Year of PICU admission**					365	<0.001
2004	12,293	*98.0*	245	*2.0*		
2005	12,326	*98.5*	193	*1.5*		
2006	12,634	*98.8*	150	*1.2*		
2007	13,275	*99.4*	84	*0.6*		
2008	13,462	*99.5*	68	*0.5*		
2009	14,023	*99.3*	100	*0.7*		
2010	14,185	*99.3*	102	*0.7*		
2011	14,006	*99.4*	90	*0.6*		
2012	14,597	*99.2*	111	*0.8*		
2013	14,865	*99.6*	57	*0.4*		
2014	14,973	*99.4*	90	*0.6*		
2015	4,028	*99.4*	26	*0.6*		

Supplemental Table 3a Random Effects Logistic Regression Model for Death in PICU (LLC only)

[n= 88,356, group = 35, wald chi2=5603, sigma_u=0.21, rho=0.02]

	Odds Ratio	95% Confidence Intervals		p-value
**Age Category**				
<1 year	REF			
1-4 years	0.85	0.78	0.92	<0.001
5-10 years	0.93	0.84	1.03	0.17
11-15 years	1.03	0.92	1.15	0.66
16+	1.37	1.12	1.67	0.002
**Sex**				
Male	REF			
Female	1.11	1.04	1.18	0.001
**Ethnicity**				
Non South Asian	REF			
South Asian	1.30	1.20	1.41	<0.001
**Deprivation Category**				
Category 1 (least deprived)	REF			
Category 2	1.00	0.88	1.13	0.96
Category 3	1.02	0.90	1.14	0.80
Category 4	1.03	0.92	1.15	0.59
Category 5 (most deprived)	1.03	0.92	1.15	0.60
**Diagnostic Group (reason for PICU admission)**				
Neurological	1.10	0.97	1.24	0.141
Cardiac	1.16	1.05	1.28	0.003
Respiratory	REF			
Oncology	1.91	1.62	2.25	<0.001
Infection	2.06	1.82	2.35	<0.001
Musculoskeletal	0.74	0.54	1.00	0.05
Gastrointestinal	1.30	1.12	1.51	<0.001
Other	1.07	0.90	1.27	0.43
Blood and lymph	2.54	1.98	3.25	<0.001
Trauma	1.13	0.73	1.74	0.58
Endocrine/metabolic	2.38	2.05	2.76	<0.001
Multisystem	0.61	0.30	1.25	0.18
Body wall and cavities	0.91	0.70	1.17	0.45
**Risk of Mortality (PIM 2)**				
<1%	REF			
1-<5%	3.86	3.29	4.53	<0.001
5-<15%	8.62	7.28	10.21	<0.001
15-<30%	18.49	15.35	22.29	<0.001
>30%	76.18	62.76	92.48	<0.001
**LOS PICU (days)**				
<1	1.47	1.33	1.62	<0.001
1 to <3	REF			
3 to <7	0.90	0.81	0.99	0.03
7 to <14	1.16	1.04	1.29	0.01

14 to <28	2.12	1.89	2.37	<0.001
>28	3.96	3.50	4.49	<0.001
**Type of PICU admission**				
Planned - after surgery	REF			
Unplanned after surgery	1.19	0.98	1.45	0.07
Planned other	1.37	1.17	1.59	<0.001
Unplanned	1.64	1.47	1.83	<0.001
Not known	1.40	0.60	3.29	0.44
**Year of Admission**	0.97	0.96	0.98	<0.001

Supplemental Table 3b Random Effects Logistic Regression Model for Death in PICU (non LLC only)

[n= 65,132, group = 35, wald chi2=4148, sigma_u=0.28, rho=0.02]

	**Odds Ratio**	**95% Confidence Intervals**		**p-value**
**Age Category**				
<1 year	REF			
1-4 years	0.66	0.56	0.77	<0.001
5-10 years	0.96	0.79	1.17	0.69
11-15 years	0.99	0.82	1.20	0.90
16+	1.08	0.60	1.92	0.80
**Sex**				
Male	REF			
Female	1.06	0.94	1.20	0.31
**Ethnicity**				
Non South Asian	REF			
South Asian	1.06	0.88	1.27	0.55
**Deprivation Category**				
Category 1 (least deprived)	REF			
Category 2	1.08	0.85	1.37	0.52
Category 3	1.02	0.81	1.28	0.88
Category 4	1.17	0.94	1.44	0.15
Category 5 (most deprived)	1.17	0.96	1.43	0.13
**Diagnostic Group (reason for PICU admission)**				
Neurological	2.19	1.79	2.69	<0.001
Cardiac	1.77	1.42	2.21	<0.001
Respiratory	REF			
Oncology	2.12	0.91	4.91	<0.001
Infection	2.05	1.63	2.58	<0.001
Musculoskeletal	0.71	0.24	2.11	0.08
Gastrointestinal	1.82	1.39	2.39	<0.001
Other	1.65	1.25	2.19	0.54
Blood and lymph	1.77	1.00	3.12	<0.001
Trauma	2.37	1.84	3.06	<0.001
Endocrine/metabolic	1.77	1.26	2.50	0.05
Multisystem	1.00			
Body wall and cavities	1.13	0.62	2.05	0.001
**Risk of Mortality (PIM 2)**				
<1%	REF			
1-<5%	7.14	4.56	11.18	<0.001
5-<15%	34.61	21.95	54.57	<0.001
15-<30%	183.20	113.88	294.69	<0.001
>30%	1530.22	954.72	2452.64	<0.001
**LOS PICU (days)**				
<1	1.66	1.43	1.93	<0.001
1 to <3	REF			
3 to <7	0.74	0.63	0.88	0.001
7 to <14	0.87	0.70	1.09	0.22
14 to <28	1.56	1.15	2.12	0.004

>28	4.07	2.75	6.02	<0.001
**Type of PICU admission**				
Planned - after surgery	REF			
Unplanned after surgery	2.04	1.36	3.08	0.001
Planned other	1.25	0.80	1.96	0.321
Unplanned	2.11	1.54	2.88	<0.001
Not known	1.83	0.32	10.33	0.494
**Year of Admission**	0.95	0.94	0.97	<0.001
